# Leptin Receptor q223r Polymorphism Influences *Clostridioides difficile* Infection-Induced Neutrophil CXCR2 Expression in an Interleukin-1β Dependent Manner

**DOI:** 10.3389/fcimb.2021.619192

**Published:** 2021-02-25

**Authors:** Olivia Horrigan, Shinsmon Jose, Anindita Mukherjee, Divya Sharma, Alexander Huber, Rajat Madan

**Affiliations:** ^1^Division of Infectious Diseases, Department of Internal Medicine, University of Cincinnati College of Medicine, Cincinnati, OH, United States; ^2^Department of Pathology and Laboratory Medicine, University of Cincinnati College of Medicine, Cincinnati, OH, United States; ^3^Division of Gastroenterology, Hepatology and Nutrition, Cincinnati Children’s Hospital Medical Center, Cincinnati, OH, United States; ^4^Veterans Affairs Medical Center, Cincinnati, OH, United States

**Keywords:** LEPR Q223R gene polymorphism, *Clostridioides (C.) difficile*, CXCR2, IL-1β, neutrophilia

## Abstract

Neutrophils are key first-responders in the innate immune response to *C. difficile* infection (CDI) and play a central role in disease pathogenesis. Studies have clearly shown that tissue neutrophil numbers need to be tightly regulated for optimal CDI outcomes: while excessive colonic neutrophilia is associated with severe CDI, neutrophil depletion also results in worse outcomes. However, the biological mechanisms that control CDI-induced neutrophilia remain poorly defined. C-X-C chemokine receptor 2 (CXCR2) is a chemotactic receptor that is critical in neutrophil mobilization from bone marrow to blood and tissue sites. We have previously reported that a single nucleotide polymorphism (SNP) in leptin receptor (LEPR), present in up to 50% of people, influenced CDI-induced neutrophil CXCR2 expression and tissue neutrophilia. Homozygosity for mutant LEPR (i.e. RR genotype) was associated with higher CXCR2 expression and more tissue neutrophils. Here, we investigated the biological mechanisms that regulate neutrophil CXCR2 expression after CDI, and the influence of host genetics on this process. Our data reveal that: a) CXCR2 plays a key role in CDI-induced neutrophil extravasation from blood to colonic tissue; b) plasma from *C. difficile*-infected mice upregulated CXCR2 on bone marrow neutrophils; c) plasma from *C. difficile*-infected RR mice induced a higher magnitude of CXCR2 upregulation and had more IL-1β; and d) IL-1β neutralization reduced CXCR2 expression on bone marrow and blood neutrophils and their subsequent accrual to colonic tissue. In sum, our data indicate that IL-1β is a key molecular mediator that communicates between gastro-intestinal tract (i.e. site of CDI) and bone marrow (i.e. primary neutrophil reservoir) and regulates the intensity of CDI-induced tissue neutrophilia by modulating CXCR2 expression. Further, our studies highlight the importance of host genetics in affecting these innate immune responses and provide novel insights into the mechanisms by which a common SNP influences CDI-induced neutrophilia.

## Introduction

*Clostridioides difficile* infection (CDI) affects more than 500,000 people in the United States annually and is classified as one of the top 5 “urgent” public-health threats (CDC, 2019). *C. difficile* causes colonic tissue damage and elicits an intense systemic and tissue immune response that is pre-dominated by neutrophils (Keel and Songer, 2006; Jose and Madan, 2016; Smits et al., 2016). Multiple studies have shown that the degree of CDI-induced neutrophilia affects clinical outcomes (El Feghaly et al., 2013a; El Feghaly et al., 2013b; Solomon et al., 2013; Na et al., 2015). In CDI patients, high peripheral blood leukocytosis, which is mainly comprised of neutrophils, is an independent risk factor for increased 30-day mortality (El Feghaly et al., 2013a). In animal models of CDI, too many colonic neutrophils are associated with severe tissue damage and high mortality (Keel and Songer, 2006), whereas neutrophil depletion is associated with higher mortality due to increased translocation of gut commensals into deeper tissues (Hasegawa et al., 2011; Jarchum et al., 2011; Jarchum et al., 2012). Therefore, neutrophil numbers need to be tightly regulated for optimal CDI outcomes. However, despite a key role for neutrophils in disease pathogenesis, the underlying biological mechanisms that regulate systemic and tissue neutrophil numbers after CDI remain poorly defined.

We have previously demonstrated that host genetic make-up impacts CDI-induced tissue neutrophilia: homozygosity for a common single nucleotide polymorphism (SNP) in leptin receptor (LEPR), rs1137101, that results in a change of amino acid at position 223 of LEPR from Glutamine [Q] to Arginine [R] (Duggal et al., 2011), is also associated with an over-exuberant neutrophil response (Jose et al., 2018a). We found that: (**i**) prior to infection, mice with the mutant/derived LEPR allele (RR) did not exhibit any obvious differences compared to those that express the wildtype allele (QQ); (**ii**) after CDI, both groups had similar pathogen burden and toxin titers, but RR mice had more systemic and tissue neutrophils; and (**iii**) RR humans with CDI had exaggerated peripheral blood leukocytosis, compared to QQ/QR (Jose et al., 2018a). Thus, CDI uncovers a hyper-inflammatory phenotype in the RR host that is manifested as exaggerated blood and colonic neutrophilia. Neutrophil numbers in systemic circulation are controlled by de-margination of already circulating cells, mobilization of mature neutrophils from the bone marrow and their efflux into tissue sites (Scheiermann et al., 2015; Christoffersson and Phillipson, 2018). Tissue accrual of neutrophils is affected by extravasation of these cells from blood and their removal at tissue sites *via* apoptosis (Furze and Rankin, 2008; Strydom and Rankin, 2013). Our previous data revealed no difference in neutrophil apoptosis after CDI between QQ and RR mice, however CXC chemokine receptor 2 (CXCR2), a neutrophil chemotactic receptor that is involved in release of mature neutrophils from bone marrow and their accumulation to sites of infection, was up-regulated in RR mice. In this study, we sought to define the biological mechanisms that promote neutrophil CXCR2 expression after CDI and the influence of LEPR Q223R SNP on these processes. We report that: (**i**) plasma from *C. difficile*-infected mice was sufficient to upregulate neutrophil CXCR2 expression; (**ii**) CDI-induced CXCR2 was upregulated only on a sub-population of neutrophils: CD11b^hi^ neutrophils; and (**iii**) Interleukin (IL)-1β was a key driver of CDI-induced neutrophil CXCR2 expression and subsequent colonic neutrophil accumulation. By utilizing mice that express the same genetic mutation as humans (RR mice), we explored the role of LEPR Q223R SNP in influencing this process. We found that: (**i**) plasma from *C. difficile*-infected RR mice resulted in a higher magnitude of neutrophil CXCR2 expression; and (**ii**) these mice had higher concentration of CDI-induced IL-1β. Since *C. difficile* is primarily a gastro-intestinal (GI) pathogen and the effects were observed in bone marrow and blood, our data suggests that IL-1β is a key intermediate in the communication between gut and bone marrow to control infection-induced neutrophilia.

## Methods

### Mouse Model of CDI

All animals were maintained and bred at the Department of Laboratory Animal Medicine, University of Cincinnati, under pathogen-free conditions in individually ventilated cages. In experiments performed to determine the effect of CXCR2 on neutrophil trafficking, C57BL/6 wildtype mice were fed with cefoperazone in drinking water and challenged with purified *C. difficile* spores (1×10^4^ VPI 10463 spores per mouse) by oral gavage as previously described (Jose et al., 2018b). CXCR2 signaling was blocked by intraperitoneal injection of SB225002 (1mg/kg/day) for 2 days prior to CDI. In experiments performed to determine the factors controlling CDI-induced neutrophilia, mice homozygous for the Q223 or R223 LEPR allele (on 129/J background) were fed with cefoperazone in drinking water and challenged with purified *C. difficile* spores (1 × 10^6^ VPI 10463 spores per mouse) by oral gavage as previously described (Jose et al., 2018a). For *in vivo* IL-1β neutralization experiments, animals were given two intraperitoneal injections of 50 µg/ml of anti-IL-1β monoclonal antibody (BioXCell #BE0246, Lebanon) each; one 24 h prior to and one at the time of infection. After infection, mice were single caged to prevent cross-infection and were monitored every 12 h. One day following infection, samples were collected for analysis.

### Neutrophil Isolation and Sorting

Bone marrow cells were harvested by flushing cleaned femur and tibia with ice cold phosphate-buffered saline containing 2% fetal bovine serum (Thermo Fisher Scientific, MA, USA) and red blood cells were lysed using ACK lysing buffer (Lonza Bioscience #10-548E, MD, USA). Cell count was determined using a TC20 automated cell counter (Bio-Rad, CA, USA). Neutrophils were then magnetically sorted using MACS MS columns (Miltenyi Biotech #130-097-658, Germany) according to the manufacturer’s instructions and cell count was determined. MACS sorted neutrophil fraction was stained with Ly6G (BioLegend #127654, CA, USA), CD11b (Thermo Fisher Scientific #12-0112-832, MA, USA), and Live/Dead stain (Thermo Fisher Scientific #L10120, MA, USA) and sorted into Roswell Park Memorial Institute (RPMI)1640 media (Thermo Fisher Scientific #1640-22400-089, MA, USA) using MoFlo FACS sorter.

### Cell Culture

Whole bone marrow or sorted neutrophils were cultured at 500,000 cells/cm^2^ in RPMI 1640 media (Thermo Fisher Scientific #1640-22400-089, MA, USA) supplemented with 10% fetal bovine serum and 1% penicillin/streptomycin. Plasma from naïve or *C. difficile*-infected QQ and RR mice stored in −80°C were thawed and added into the culture media at 1% concentration and cells were incubated at 37°C in a 5% CO_2_ incubator for designated time period (6–24 h). Recombinant IL-1β (1 µg/ml), and anti-IL-1β (5 µg/ml) neutralizing antibodies were added along with plasma to deduce the role of these cytokines in regulation of CXCR2 expression. In some experiments, plasma samples were treated with heat (at 56°C for 15 min) or Proteinase-K (4 µg/ml at 37°C for 1 h) before adding into culture media to eliminate complement and total proteins respectively.

In separate experiments to examine transcriptional regulation of CXCR2, whole bone marrow cells were cultured in RPMI media with IL-1β for 18 h and neutrophils were sorted by flow cytometry. RNA extracted from flow sorted neutrophils using Qiagen RNeasy Plus Mini kit, and used to examine CXCR2 mRNA expression by qPCR (TaqMan gene expression assay; ThermoFisher; Cat# 4331182). Paired unstimulated cells were used as controls.

### Flow Cytometry

Cells harvested after stipulated culture period were stained with fixable Live/Dead dye (Thermo Fisher Scientific; #L10120; MA, USA) for 15 min at 4°C in a 96 well plate. Cells were washed twice and blocked with unlabeled anti-CD16/32 (Fc-block; BD Biosciences). After blocking, cells were stained with pre-titrated fluorescently labeled antibodies (Ly6G, CD11b and CXCR2; **Supplementary Table 1**) in FACS buffer (2% fetal bovine serum in PBS) for 30 min at 4°C. Cells were washed to remove excess antibodies and suspended in FACS buffer. Flow cytometry was performed on a BD FACS Accuri C6 flow (BD Biosciences) cytometer equipped with CFlow Plus (BD Biosciences) software and analyzed using FlowJo (Treestar). Gating was performed based on fluorescence-minus one (FMO) controls (**Supplementary Figure 5**).

### ELISA

Plasma was separated from blood samples by centrifugation at 5,000x g at 4°C for 5 min and stored at −80°C until use. Cecal tissue samples were rinsed gently in PBS to remove cecal contents and frozen immediately by immersing in liquid nitrogen. Lysates were prepared in RIPA buffer using gentleMACS tissue dissociator (Miltenyi Biotec, CA, USA). Manufacturer defined program for homogenization of intestine was used to prepare the lysates. Homogenate was centrifuged at 13,000 x g for 5 min and supernatant was collected and stored at −80°C for further use. Levels of IL-1β in plasma and tissue lysates were measured by ELISA using a protocol provided by the manufacturer (BioLegend, USA).

### Histopathology of Cecal Tissue Sections

Cecal tissue samples were fixed in Bouin’s solution (Sigma) overnight. Samples were washed and dehydrated in 70% ethanol prior to paraffin embedding. Four-micron sections were stained with hematoxylin and eosin (H&E) and scored for inflammatory cell infiltration, edema and epithelial disruption. A score of 0 to 4, denoting increasingly severe abnormality, was assigned for each of these parameters by a pathologist in a blinded fashion.

### Oxidative Burst and Phagocytosis Assays

Oxidative burst and phagocytic capacity of CD11b^hi^ and CD11b^low^ tissue neutrophils were examined as described previously (Jose et al., 2018a). To evaluate ROS generation, purified neutrophils were stimulated with N-Formylmethionyl-leucyl-phenylalanine (fMLP) in presence of Dihydrorhodamine (DHR-123), and fluorescence generated by the oxidation of DHR into fluorescent rhodamine-123 was enumerated by flow cytometry. To evaluate phagocytic ability, purified tissue neutrophils were incubated with opsonized *E.coli* bioparticles conjugated with AF-488 (Molecular probes E-2870) in RPMI medium. Cells were washed with ice-cold PBS. Mean fluorescent intensity of AF-488 was estimated by flow cytometry.

### Statistical Methods

All statistical analyses were performed using GraphPad Prism 5.0 software (GraphPad software Corporation, Inc, CA, USA). For comparison of groups, a Student’s t-test or ANOVA with Tukey’s post-hoc test was used. A p value below 0.05 was considered significant.

## Results

### CXCR2 Antagonism Reduces CDI-Induced Tissue Neutrophilia Without Effects on Blood Neutrophils

**Figure 1:**
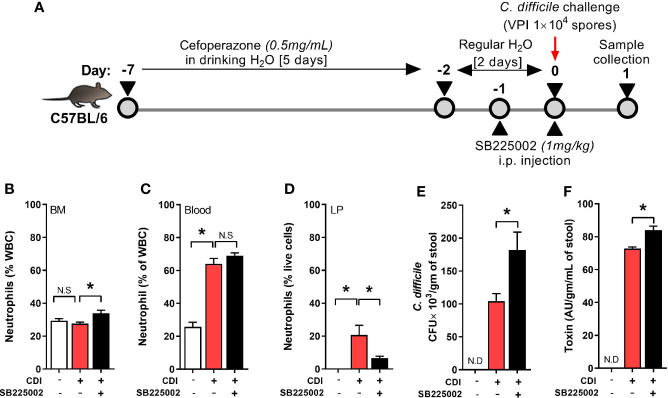
CXCR2 blocking reduced *C*. *difficile* infection (CDI)-induced colonic neutrophilia. **(A)** Experimental design of CDI and CXCR2 blocking. Wildtype C57Bl/6 mice were pre-treated with antibiotics for 5 days in drinking water, followed by 2 days of regular water and then challenged with 1 × 10^4^ *C. difficile* (VPI 10463) spores by oro-gastric gavage. Mice were treated with selective CXCR2 antagonist (SB225002, 1 mg/kg/mouse by intra-peritoneal injection). Animals were sacrificed on day 1 after infection to evaluate neutrophil dynamics, using Hemavet and fluorescent activated cell sorting (FACS) analysis. **(B–D)** Plots show proportion of neutrophils in **(B)** Bone marrow (BM), **(C)** Blood; and **(D)** Lamina propria (LP). **(E, F)** *C*. *difficile* **(E)** bacterial colony forming unit (CFU); and **(F)** toxin A/B per gram of cecal content of *C. difficile*-challenged mice. Data shown as mean ± s.e.m. n=6 per group; *p < 0.05, Student’s t-test. N.S, non-significant.

Neutrophil trafficking is regulated *via* CXC chemokines and their respective receptors (Eash et al., 2010). Binding of CXC ligands (CXCL 1 and 2) to CXCR2 promotes release of neutrophils from bone marrow and their homing to inflamed tissue (Strydom and Rankin, 2013). CDI induces both CXCL 1 and 2, but their plasma and tissue concentrations are similar between QQ and RR mice (Jose et al., 2018a). However, RR mice had higher CXCR2 expression on blood and bone marrow neutrophils (Jose et al., 2018a) suggesting that augmented receptor upregulation in this group contributes to the increased systemic and tissue neutrophilia after CDI. To directly test the role of CXCR2 in regulating neutrophil trafficking in response to CDI, we treated wildtype mice with a selective CXCR2 antagonist (SB225002 1 mg/kg i.p.) or control (PBS) for 2 days prior to CDI (**Figure 1A**) (White et al., 1998; Bento et al., 2008; Zhu et al., 2020). On day 1 after infection, neutrophils in bone marrow, blood and colonic tissue were quantified. Compared to those treated with PBS, SB225002-treated *C. difficile* infected mice had a slight increase in the proportion of neutrophils in bone marrow (**Figure 1B**) and no effect on blood neutrophils (**Figure 1C**). However, CXCR2 antagonism significantly reduced CDI-induced neutrophil accrual in the colonic lamina propria (**Figure 1D**). These data indicate a key role for CXCR2 in mobilization of neutrophils from blood to tissue sites. SB225002 treatment resulted in increase of *C. difficile* bacteria and toxin titers in intra-cecal contents (**Figures 1E, F**).

### CDI Enhances CXCR2 Expression on CD11b^hi^ Neutrophil Sub-Population in Blood

**Figure 2:**
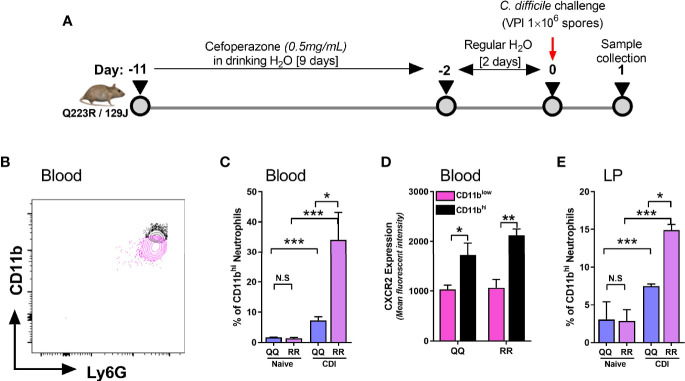
CD11b^hi^ blood neutrophils express CXCR2 after *C. difficile* infection (CDI). **(A)** Schematic representation of experimental design of *C. difficile* infection in QQ and RR mice. Age- and gender- matched QQ and RR mice were pre-treated with antibiotics for 9 days in drinking water, followed by 2 days of regular water and then challenged with 1x10^6^ *C*. *difficile* (VPI 10463) spores by oro-gastric gavage. Mice were sacrificed on day 1 after infection to evaluate neutrophil CD11b expression in **(B–D)** blood and **(E)** lamina propria. **(B)** Representative fluorescent activated cell sorting (FACS) plot showing CD11b^hi^ and CD11b^low^ neutrophils. **(C)** Proportion of CD11b^hi^ neutrophils in blood of naïve and *C. difficile* infected QQ and RR mice. **(D)** Mean fluorescence intensity (MFI) of CXCR2 expression on CD11b^hi^ and CD11b^low^ neutrophil sub-populations in blood of *C*. *difficile* infected QQ and RR mice. **(E)** Proportion of total lamina propria neutrophils that are CD11b^hi^ in naïve and *C*. *difficile*-infected QQ and RR mice. Data shown as mean ± s.e.m. of n=3-4; representative of 3 independent experiments; ***p < 0.001, **p < 0.01, *p < 0.05, Student’s t-test. N.S, non-significant.

CXCR2 is a G-protein coupled receptor (GPCR) that promotes leukocyte mobilization by activation of integrin molecules on cell surface (Cacalano et al., 1994; Zarbock et al., 2012; Zarbock and Stadtmann, 2012). Integrins are hetero-dimers comprised of paired α and β chains that mediate leukocyte-endothelium interactions and participate in extravasation of neutrophils from blood vessels to inflamed tissue sites (Zarbock et al., 2012). CD11b is a β2 integrin present on all neutrophils and its expression is upregulated during the innate host response to various noxious stimuli (O’Hare et al., 2015; Hesselink et al., 2019; Stockfelt et al., 2020). CD11b dimerizes with CD18 (common α chain of various integrins) to promote neutrophil adhesion to endothelial cells and their trans-endothelial migration into tissue sites (Zarbock and Stadtmann, 2012; Arnaout, 2016). We examined neutrophil CD11b and CXCR2 expression in wildtype (i.e. QQ) and LEPR mutant (i.e. RR) mice on day 1 after CDI (**Figure 2A**). Since RR mice are on 129J background, we utilized a longer course of antibiotics and higher dose of *C. difficile* spores, as per our previously published protocol (Jose et al., 2018a). Based on mean fluorescent intensity of CD11b, we detected two distinct neutrophil populations in blood of *C. difficile*-infected mice (**Figure 2B**). Uninfected QQ and RR mice had very few CD11b^hi^ neutrophils, but after infection their proportion increased in both groups (**Figure 2C**). Among the two groups, RR mice had significantly higher CD11b^hi^ neutrophils, compared to QQ mice (**Figure 2C**). Notably, CDI-induced CXCR2 expression was upregulated only on CD11b^hi^ neutrophil sub-population but not on the CD11b^low^ sub-population (**Figure 2D**). These data suggest that CD11b^hi^ cells are primed for extravasation to tissue sites. Indeed, CDI resulted in increased proportion of CD11b^hi^ neutrophils in the lamina propria, and again RR mice had more of these cells (**Figure 2E**). In addition, we found that these CD11b^hi^ neutrophils are highly activated in nature: phagocytosis of *E.coli* bioparticles and the amount of reactive oxygen species (ROS) generated in response to *ex vivo* N-Formyl methionyl-leucyl-phenylalanine (fMLP) stimulation was higher in CD11b^hi^ neutrophils, compared to CD11b^low^ neutrophils (**Supplementary Figure 1**).

### Plasma From *C. difficile*-Infected Mice Upregulates Neutrophil CXCR2 Expression *In Vitro*

**Figure 3:**
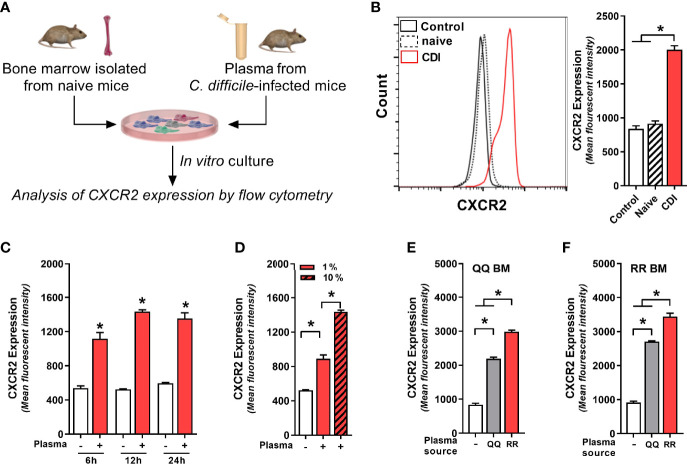
Plasma from *C*. *difficile*-infected mice induces neutrophil CXCR2 expression. **(A)** Experimental design. Whole bone marrow cells from **(B–E)** wildtype (i.e. QQ) mice and **(F)** RR mice were cultured in Roswell Park Memorial Institute (RPMI) media with no plasma (control) or plasma collected from uninfected (naïve) and *C. difficile* infected (CDI) RR mice at 1% v/v. CDI-plasma was collected on day 1 after infection and stored at −80°C till use in cultures. Surface expression of CXCR2 protein on neutrophils was analyzed by flow cytometry at different time points. **(B)** Representative fluorescent activated cell sorting (FACS) histogram and mean fluorescence intensity (MFI) of CXCR2 expression on neutrophils 24 h of culture. **(C)** CXCR2 expression at 6 h, 12 h, and 24 h of culture. **(D)** CXCR2 expression at 24 h of culture in the presence of 1% and 10% CDI-plasma. CDI-plasma induced CXCR2 on neutrophils in whole bone marrow cultures of **(E)** QQ mice and **(F)** RR mice. Data shown as mean ± s.e.m. of n=4–5; representative of three independent experiments; *p < 0.05, Student’s t-test or ANOVA with Tukey’s post-hoc test. N.S, non-significant.

Although *C. difficile* primarily resides in the GI tract, multiple studies have reported communication between gut and bone marrow *via* mediators secreted in systemic circulation (Santisteban et al., 2016; Wang et al., 2019; Burgess et al., 2020). Thus, we postulated that CDI-induced factors from GI tract are released into plasma and they act as intermediaries in the gut-bone marrow axis to regulate neutrophil CXCR2 expression. To test this hypothesis, we cultured freshly isolated bone marrow cells from wildtype mice (i.e. QQ) with no plasma (control), plasma from uninfected mice (naïve) or plasma from *C. difficile-*infected wildtype mice (CDI-plasma; **Figure 3A**). Since CXCR2 upregulation and neutrophil recruitment after CDI was observed 1 day after infection (**Figures 1** and **2**), for our *in vitro* experiments we utilized plasma collected from the same time-point. Our data show that bone marrow cells cultured with CDI-plasma had higher CXCR2 expression, compared to those cultured without plasma or those with plasma from naïve mice (**Figure 3B**). While CXCR2 is present on multiple different cell types, the CDI plasma-induced increase was seen only on neutrophils (i.e. CD11b^+^Ly6G^+^ cells), whereas non-neutrophil bone marrow cells (i.e. CD11b^+^Ly6G^-^ cells) did not exhibit CXCR2 up-regulation (**Supplementary Figure 2A**). Increase in CXCR2 MFI was observed within 6 h, with highest expression observed after 12 h of incubation (**Figure 3C**).

Although neutrophils are believed to have a relatively short lifespan, in cases of infection and inflammation they can survive for longer periods of time (Hidalgo et al., 2019). Consistent with this observation, cells that were cultured in presence of CDI-plasma had longer survival and <5% cell death was seen in neutrophils at 24 h (**Supplementary Figure 2B**). Cells cultured without plasma or with plasma from uninfected mice (naïve), on the other hand, exhibited a decline in neutrophil viability after 12 h and ~35% neutrophils were dead by 24 h (**Supplementary Figure 2B**). The increase in CXCR2 MFI was dependent on amount of plasma used: cells incubated with higher concentration of CDI-plasma (10% v/v) had more CXCR2, compared to those incubated with low concentration (1% v/v) (3.76 ± 0.21 vs 2.96 ± 0.04 fold increase in MFI; **Figure 3D**). The intensity of CXCR2 MFI was independent of the bone marrow source, but it was dependent on plasma source: cells incubated with CDI-plasma collected from RR mice had higher CXCR2, compared to those incubated with plasma from *C. difficile*-infected QQ mice (**Figures 3E, F**). Our data thus indicate that CDI-induced plasma factor(s) support neutrophil survival *in vitro* and upregulate neutrophil CXCR2 expression in a dose-dependent manner. In addition, the concentration of such factor(s) is likely to be higher in the plasma of *C. difficile*-infected RR mice.

### CDI-Induced Protein Component in Plasma Upregulates Neutrophil CXCR2 Expression

**Figure 4:**
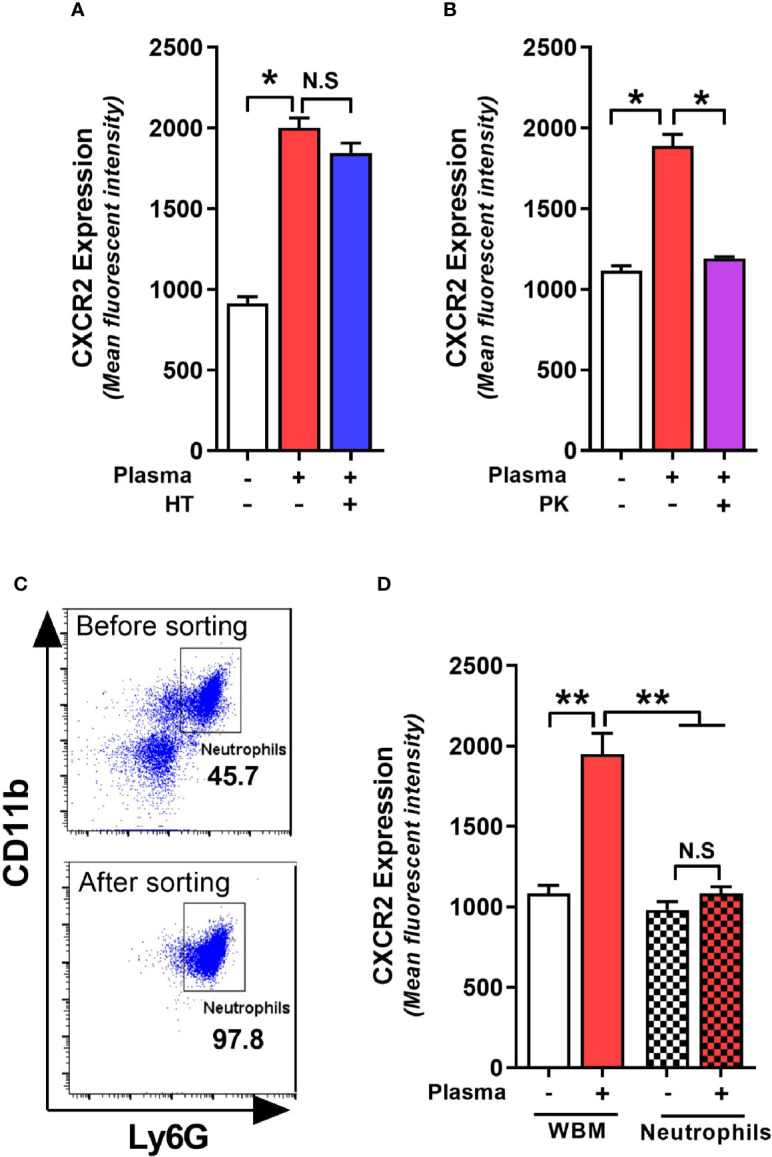
*C*. *difficile* infection (CDI)-plasma induced neutrophil CXCR2 upregulation is dependent on protein component of plasma and requires the presence of non-neutrophil bone marrow cells. Whole bone marrow cells and fluorescent activated cell sorting (FACS)-purified neutrophils from wildtype (i.e. QQ) mice were cultured in Roswell Park Memorial Institute (RPMI) media with CDI-plasma from RR mice (1% v/v). Mean fluorescence intensity (MFI) of CXCR2 expression on neutrophils at 24 h in the presence of **(A)** heat treated (i.e. complement-deficient), and **(B)** proteinase K-treated (i.e. with all proteins degraded) plasma. **(C)** Representative FACS dot plot showing neutrophil percentage before and after sorting. **(D)** Neutrophil CXCR2 expression at 24 h in whole bone marrow and purified neutrophil cultures. Data shown as mean ± s.e.m of n=3-4; representative of 2–3 independent experiments; *p < 0.05, **p < 0.01, Student’s t-test. N.S, non-significant.

Plasma from *C. difficile*-infected RR mice resulted in maximal upregulation of CXCR2 and MFI increase was similar in bone marrow cells collected from both QQ and RR mice (**Figures 3E, F**). Therefore, in subsequent experiments we utilized CDI-plasma from RR mice and bone marrow cells from QQ mice to identify the putative CXCR2-inducing factor(s). The plasma portion of blood contains an array of proteins, hormones, electrolytes and microbial metabolites (Atkins et al., 2017). Complement constitutes a system of distinct proteins found in blood plasma and is known to antagonize CXCR2-mediated neutrophil mobilization (Brennan et al., 2019). We first tested the role of complement in upregulating neutrophil CXCR2 by incubating wildtype bone marrow cells with heat-treated (i.e. complement inactivated) CDI-plasma. There was no difference in CXCR2 MFI on neutrophils exposed to control or heat-treated plasma (**Figure 4A**). To identify if proteins other than complement can affect CXCR2 expression, we degraded all plasma proteins in CDI-plasma by proteinase K treatment prior to incubation with bone marrow cells. Bone marrow cells cultured with proteinase K-treated CDI-plasma did not exhibit neutrophil CXCR2 upregulation (**Figure 4B**). These data suggest that a protein component of plasma, different from complement, is responsible for up-regulating neutrophil CXCR2.

Whole bone marrow consists of a mixture of neutrophils and non-neutrophil cell populations. Therefore, the effects of plasma from *C. difficile*-infected mice on CXCR2 expression could result from direct action on neutrophils or indirectly *via* effects on other bone marrow cells. To test if the effects of CDI-plasma on CXCR2 upregulation is neutrophil intrinsic or extrinsic, we used FACS-purified neutrophils (>97% pure CD11b^+^Ly6G^+^ cells; **Figure 4C**) from wildtype mice in *in vitro* experiments. While incubation with CDI-plasma increased neutrophil CXCR2 MFI in whole bone marrow cultures, there was no increase seen when only purified neutrophils were used (**Figure 4D**), indicating that the effect of plasma from *C. difficile*-infected mice on the induction of neutrophil CXCR2 requires the presence of other bone marrow cells.

### RR Mice Have Higher IL-1β Concentration After Infection and IL-1β Is a Key Driver of CDI-Induced Neutrophil CXCR2 Expression

**Figure 5:**
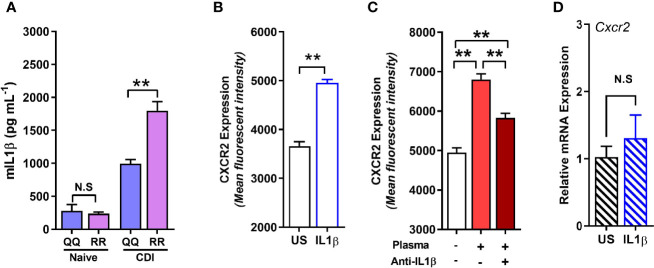
RR mice have higher *C*. *difficile* infection (CDI)-induced interleukin (IL)-1β production, and IL-1β upregulates neutrophil CXCR2 *in vitro*. **(A)** Concentration of IL-1β in plasma of naïve and *C. difficile*-infected mice (day 1 after infection). **(B, C)** Mean fluorescence intensity (MFI) of CXCR2 expression on neutrophils in whole bone marrow cultures from wildtype (i.e. QQ) mice at 24 h in the presence of **(B)** recombinant IL-1β; and **(C)** CDI-plasma from RR mice (1% v/v) in the presence or absence of anti-IL-1β antibody (5 µg/ml). **(D)** Relative CXCR2 mRNA expression in neutrophils sorted from unstimulated (US) and IL-1β- stimulated whole bone marrow cultures. Data shown as mean ± s.e.m. n=4–5; representative of 2–3 independent experiments; *p < 0.05, **p < 0.01, Student’s t-test. N.S, non-significant.

**Figure 6:**
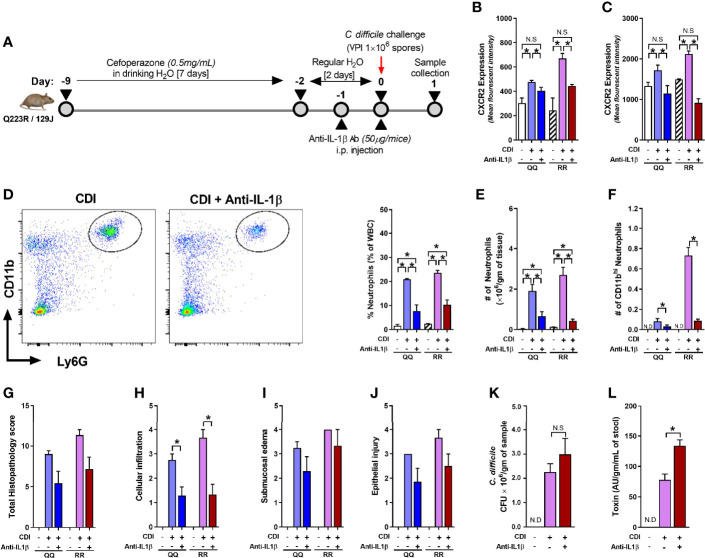
Interleukin (IL)-1β neutralization blocks *C*. *difficile* infection (CDI)-induced tissue neutrophil accrual. **(A)** Schematic representation of experimental design of *C. difficile* infection in QQ and RR mice. Age- and gender- matched QQ and RR mice were pre-treated with antibiotics for 9 days in drinking water, followed by 2 days of regular water and then challenged with 1 × 10^6^ *C. difficile* (VPI 10463) spores by oro-gastric gavage. Mice were treated with anti-IL-1β antibody (1 µg/mouse by intra-peritoneal injection), and samples were collected 1 day after infection. Mean fluorescence intensity (MFI) of CXCR2 expression on **(B)** bone marrow neutrophils and **(C)** blood CD11b^hi^ neutrophils. **(D)** Percentage lamina propria neutrophils in *C*. *difficile*-infected mice with or without anti-IL-1β antibody. Number of **(E)** total and **(F)** CD11b^hi^ neutrophils in lamina propria of *C*. *difficile*-infected mice with or without anti-IL-1β antibody. **(G)** Total histology score for **(H)** submucosal edema, **(I)** epithelial injury, and **(J)** inflammatory cell infiltration in cecal tissue sections. *C. difficile* **(K)** bacterial colony forming unit (CFU), and **(L)** toxin A/B per gram of cecal content of *C*. *difficile*-challenged mice. Data shown as mean ± s.e.m of n=3–8 per group; representative of two independent experiments; *p < 0.05, **p < 0.01, ***p < 0.001; Student’s t- test. N.S, non-significant; N.D, not detected.

Although concentration of several cytokines and chemokines that affect CXCR2 surface expression (e.g. G-CSF, GMCSF, CXCL-1, and CXCL-2) was not different between *C. difficile*-infected QQ and RR mice (Jose et al., 2018a), we found that RR mice had higher plasma concentrations of the prototypic pro-inflammatory cytokine Interleukin (IL)-1β (**Figure 5A**). IL-1β is implicated in host resistance to acute bacterial infections (Miller et al., 2007; Rider et al., 2011). In CDI pathogenesis, reduced IL-1β during acute infection was associated with fewer colonic neutrophils (Hasegawa et al., 2012). In colonic tissue, CDI induced both IL-1β mRNA and protein expression in QQ and RR mice (**Supplementary Figures 3A, B**). While there was no difference in IL-1β mRNA between the two strains, RR mice had higher protein expression (**Supplementary Figures 3A, B**). We next examined the role of IL-1β in controlling neutrophil CXCR2 expression by manipulating its level *in vitro* and *in vivo*. *In vitro*, incubation of bone marrow cells from wildtype mice with recombinant IL-1β resulted in a significant increase in neutrophil CXCR2 (**Figure 5B**), whereas neutralizing IL-1β with anti-IL-1β antibody in CDI-plasma reduced the increase in neutrophil CXCR2 (**Figure 5C**). Published literature shows that CXCR2 expression on cell surface can be regulated at the level of gene transcription and/or post-transcriptionally *via* mechanisms that include receptor internalization/recycling and cleavage by proteases (Neel et al., 2005; Mishra et al., 2015). We found that *Cxcr2* mRNA expression in neutrophils sorted from whole bone marrow cultures treated with IL-1β and was similar to that from untreated controls (**Figure 5D**). Taken together, these *in vitro* experiments suggest that IL-1β upregulates the surface expression of CXCR2 at a post-transcriptional level. *In vivo*, treatment of mice with anti-IL-1β antibody prior to infection (**Figure 6A**), abolished the increase in IL-1β that was seen after CDI in both plasma and colonic tissue of QQ and RR mice (**Supplementary Figures 3B, C**). IL-1β neutralization reduced CXCR2 MFI to pre-infection levels on total and CD11b^hi^ blood neutrophils in both QQ and RR mice (**Figures 6B, C**). Consistent with decreased neutrophil CXCR2, the percentage of total neutrophils and the number of total and CD11b^hi^ neutrophils in lamina propria was reduced in all *C. difficile*-infected mice that received anti-IL-1β antibody (**Figures 6D–F**). Histology scoring of cecal tissue sections also showed effects of IL-1β neutralization on cellular infiltration: anti-IL-1β treated mice had lower cellular infiltration score, but submucosal edema and epithelial damage score was similar to controls (**Figures 6G–J**; **Supplementary Figure 4**). Although IL-1β neutralization did not have any effect on *C. difficile* pathogen (**Figure 6K**), toxin titers were elevated in mice with IL-1β blocking (**Figure 6L**). In sum, our data illustrate a critical role for CDI-induced IL-1β in upregulating neutrophil CXCR2 and their subsequent trafficking to the site of infection. Further, the magnitude of CDI-induced increase in IL-1β, CXCR2 expression and the subsequent tissue neutrophilia was influenced by host SNP type with RR mice exhibiting an increase in all these parameters.

## Discussion

Neutrophils are key first-responders in the innate immune response to a wide range of infectious insults (Drescher and Bai, 2013; Nauseef and Borregaard, 2014). Bone marrow is the primary reservoir for mature neutrophils and after infectious insults these cells are rapidly released to blood and then mobilized to inflamed tissues (Furze and Rankin, 2008; Delano et al., 2011; Lawrence et al., 2018). While neutrophils are essential for host defense, their excessive numbers and/or aberrant activation can result in collateral host damage (Nauseef and Borregaard, 2014; Silvestre-Roig et al., 2016). At the same time, lack of neutrophils can result in pathogen-mediated damage (Viscoli et al., 2005). After CDI, both excessive tissue neutrophilia as well as neutropenia are associated with poor host survival (Smith, 1994; Solomon et al., 2013; Farooq et al., 2015; Jose et al., 2018a). Therefore, it is important to understand the mechanisms that regulate infection-induced neutrophil responses and therefore result in optimal disease outcomes. We have previously shown a key role for host genetics in influencing CDI-induced CXCR2 expression and tissue neutrophilia (Jose et al., 2018a). Here, we utilized mice with the same SNP to interrogate the mechanism(s) of CXCR2 upregulation after CDI. The most important findings of our study are: **i)** CXCR2 is a critical driver of tissue neutrophilia after CDI; **ii)** IL-1β is a key molecular mediator in the gut-bone marrow axis that promotes CXCR2 upregulation and CDI-induced colonic neutrophil accrual; and **iii)** IL-1β concentration is differentially regulated based on host SNP type.

Our data clearly show that presence of mutant LEPR allele resulted in more IL-1β and tissue neutrophils during acute CDI, despite similar amounts of *C. difficile* pathogen burden in the two strains of mice (Jose et al., 2018a). Although the SNP-associated differences in tissue neutrophil numbers were lost after IL-1β neutralization (**Figures 6E, F**), this is not totally unexpected since the antibody dose used in our studies results in significant depletion of IL-1β and brings the concentration down to pre-infection levels in both QQ and RR mice (**Supplementary Figure 3B, C**). Notably, the SNP-associated differential effect on IL-1β was seen only at the protein level: tissue mRNA expression of IL-1β on day 1 of CDI was similar in QQ and RR mice (**Supplementary Figure 3A**) but IL-1β protein was higher in both tissue lysates and in plasma of *C. difficile*-infected RR mice. IL-1β is expressed in its precursor form, pro-IL1β, which then undergoes post-translational cleavage by caspase-1 to release mature IL-1β. Caspase-1 activation is facilitated by NLRP3 inflammasome (Schroder and Tschopp, 2010; Dinarello et al., 2012) and this inflammasome-induced IL-1β is crucial for CDI-induced neutrophil recruitment (Hasegawa et al., 2012; Liu et al., 2018). Since ELISA detects both pro- and mature- IL-1β, tissue lysates could represent a combination of both forms of IL-1β, but increase in plasma likely reflects only the mature form and suggests a role for inflammasome activation in this process. An important mechanism of NLRP3 inflammasome activation is mediated by caspase-3 (Vince et al., 2018), and RR mice have high caspase-3 in intestinal epithelial cell after *Entamoeba histolytica* infection (Duggal et al., 2011). It is noteworthy that RR individuals have worse outcomes in sepsis and after *E. histolytica* infection (Bracho-Riquelme et al., 2011; Duggal et al., 2011). While the exact mechanisms that result in worse disease in an RR host remain undefined, our data along with the published studies raises the intriguing possibility that LEPR SNP-type associated effects on the inflammasome pathway could elevate IL-1β which drives a hyper-inflammatory phenotype in an RR host and contributes to poor infectious disease outcomes. Future experiments will be aimed at studying the impact of LEPR Q to R change on CDI-induced inflammasome activation and subsequent IL-1β processing and release.

*C. difficile* is primarily a colonic pathogen, but CDI affects neutrophil homeostasis at distant sites (e.g. bone marrow). Our data suggest that IL-1β is a crucial mediator in the gut-bone marrow axis that promotes CXCR2 expression and CDI-induced neutrophilia. However, the role of gut microbiome in this process remains unknown at this time. Dysbiosis is central in CDI pathogenesis and gut microbiota clearly regulates both CDI-induced IL-1β generation and bone marrow neutrophil CXCR2 expression (Hasegawa et al., 2012; Deshmukh et al., 2014; Watanabe et al., 2017; Jose et al., 2018a). In particular, IL-1β was produced in *C. difficile*-infected mice only in the presence of γ-proteobacteria after their translocation from colonic lumen to deeper tissues (Hasegawa et al., 2012). We have previously shown that the relative abundance of proteobacteria was higher in RR mice that also have more IL-1β during acute CDI. Reciprocal transfer of gut microbiota between QQ and RR mice prior to infection abolished the difference in CDI-induced CXCR2 expression between the two groups (Jose et al., 2018a). Together, these observations support a potential role for antibiotic and CDI-induced alterations in gut microbiota, which is differentially regulated based on host genetics and then affects IL-1β expression. Another important observation from our studies is that non-neutrophil bone marrow cells are essential for CXCR2 expression on neutrophil surface. Bone marrow consists of a mixture of cells that include hematopoietic, mesenchymal stromal and other niche cells. A role for non-hematopoietic cells in controlling the generation and mobilization of neutrophils after noxious insults has been previously reported (Ueda et al., 2009; Boettcher et al., 2012; Burberry et al., 2014). Expression of the receptor for IL-1β (i.e. IL-1R1) on radiation-resistant bone marrow cells that are mainly comprised of mesenchymal cells is critical for generation and release of neutrophils from bone marrow (Ueda et al., 2009). Thus, our data, along with published literature, suggest a hypothesis that IL-1β acts on stromal cells to influence neutrophil CXCR2 expression. CXCR2 can be regulated at the level of transcription and/or post-transcriptionally *via* receptor internalization/recycling and cleavage at the cell surface (Fan et al., 2003; Neel et al., 2005; Mishra et al., 2015). Our data shows that exogenous IL-1β on whole bone marrow does not induce CXCR2 mRNA expression in neutrophils (**Figure 5D**). Therefore, the most likely mechanism by which IL-1β affects neutrophil CXCR2 expression is at the protein level. However, more studies are clearly needed to: (i) define the perturbations in gut microbiota that affect IL-1β production after CDI; (ii) identify the bone marrow cells that respond to this cytokine to promote CXCR2 induction; and (iii) examine the role of post-transcriptional modifications (i.e. receptor internalization/recycling and surface shedding) by which IL-1β enhances CXCR2 expression.

Although presence of morphologically and functionally distinct neutrophil populations have been reported after other bacterial infections (Tsuda et al., 2004; Hellebrekers et al., 2017; Deniset and Kubes, 2018), there are no studies that have investigated neutrophil heterogeneity after CDI. Our data shows for the first time that distinct neutrophil sub-populations, distinguished by CD11b MFI, were present in blood of *C. difficile*-infected mice. Of the two neutrophil sub-populations identified, only CD11b^hi^ neutrophils exhibited an increase in CDI-induced CXCR2 expression and these cells accumulated in higher numbers in the colon of RR mice. Therefore, these cells seem to be primed for mobilization from bloodstream to tissue sites. CD11b is a marker of neutrophil activation and is instrumental in the release of intra-cellular mediators that can be toxic to host cells (Conejeros et al., 2011; Kelm et al., 2020). Indeed, the CD11b^hi^ neutrophils present in *C. difficile*-infected mice generated more ROS and phagocytosed more *E. coli* beads compared to CD11b^low^ cells. However, after neutrophil extravasation into colonic lumen, CD11b binding to epithelial cells can also promote mucosal healing (Sumagin et al., 2016). We have previously observed that despite similar pathogen load or *C. difficile* toxin titers in QQ and RR mice, the latter group had more colonic tissue damage (Jose et al., 2018a). But, RR mice that survived the initial insult had improved histology scores and faster disease resolution later on (Jose et al., 2018a). The dichotomous nature of CD11b actions could potentially explain this phenotype, where too many CD11b^hi^ neutrophils in lamina propria during early CDI are harmful, but later on their presence in colonic lumen helps with mucosal healing and repair.

In sum, our studies support a scenario where CDI-induced IL-1β acts *via* CXCR2 to regulate CDI-induced tissue neutrophilia. Our data further illustrate a crucial role for host genetic make-up in influencing IL-1β concentration. Since this SNP is present in up to 50% of individuals and infection-induced neutrophilia is a fundamental biological process, our study has implication beyond just CDI, as this SNP can serve as a valuable genetic biomarker to predict innate immune responses after various different infectious insults.

## Data Availability Statement

The original contributions presented in the study are included in the article/**Supplementary Material**. Further inquiries can be directed to the corresponding authors.

## Ethics Statement

The animal study was reviewed and approved by Institutional Animal Care and Use Committee, University of Cincinnati.

## Author Contributions

SJ and RM conceptualized and designed the study. OH conducted *in vitro* experiments and flow cytometry. SJ and AM performed animal studies. AH performed ELISA assays. DS reviewed and scored histopathology slides. OH, AH, SJ, and RM wrote the manuscript. RM obtained funding for the project. All authors contributed to the article and approved the submitted version.

## Funding

This work was supported in part by the National Institutes of Health (NIH) K08-AI108801-01 and Veterans Affairs MERIT Review I01BX004630 (both to RM), and by PHS Grant P30 DK078392 (Digestive Diseases Research Core Center in Cincinnati).

## Conflict of Interest

The authors declare that the research was conducted in the absence of any commercial or financial relationships that could be construed as a potential conflict of interest.

## Supplementary Material

Supplementary Figure 1 Flow cytometric analysis of **(A, B)** phagocytosis (*E. coli* AF-488) and **(C, D)** oxidative burst; DHR-123 in tissue neutrophils after *ex vivo* fMLP stimulation. n=4; representative of 2 independent experiments, data shown as mean ± s.e.m; *p<0.05, Student’s t-test.

Supplementary Figure 2 **(A)** CXCR2 expression on CD11b^+^Ly6G^-^ cells of bone marrow after 24h of *in vitro* culture in presence of plasma from *C. difficile*-infected RR mice. **(B)** Percent live neutrophils at 12h and 24h of *in vitro* culture. n=4-5; representative of 2 independent experiments, data shown as mean ± s.e.m; *p<0.05, Student’s t-test.

Supplementary Figure 3 **(A)** mRNA expression IL-1β in cecal tissue of naïve and *C. difficile*-infected (day 1) 129J QQ and RR mice. IL-1β protein concentration in **(B)** colonic tissue and **(C)** blood in QQ and RR mice after CDI and with and without anti-IL-1β antibody treatment. n=6-10 mice/group; data shown as mean ± s.e.m; *p<0.05, Student’s t-test.

Supplementary Figure 4 Representative histology images of hematoxylin and eosin–stained cecal tissue sections of QQ and RR mice at day 1 of *C. difficile* infection.

Supplementary Figure 5 Gating strategy for flow cytometry analysis of neutrophils.
