# Supplementary figures and images for: Leptin Receptor q223r Polymorphism Influences Clostridioides difficile Infection-Induced Neutrophil CXCR2 Expression in an Interleukin-1β Dependent Manner
Source: Front Cell Infect Microbiol. 2021 Feb 25;11:619192. doi: 10.3389/fcimb.2021.619192 (PMC7946998; doi:10.3389/fcimb.2021.619192)

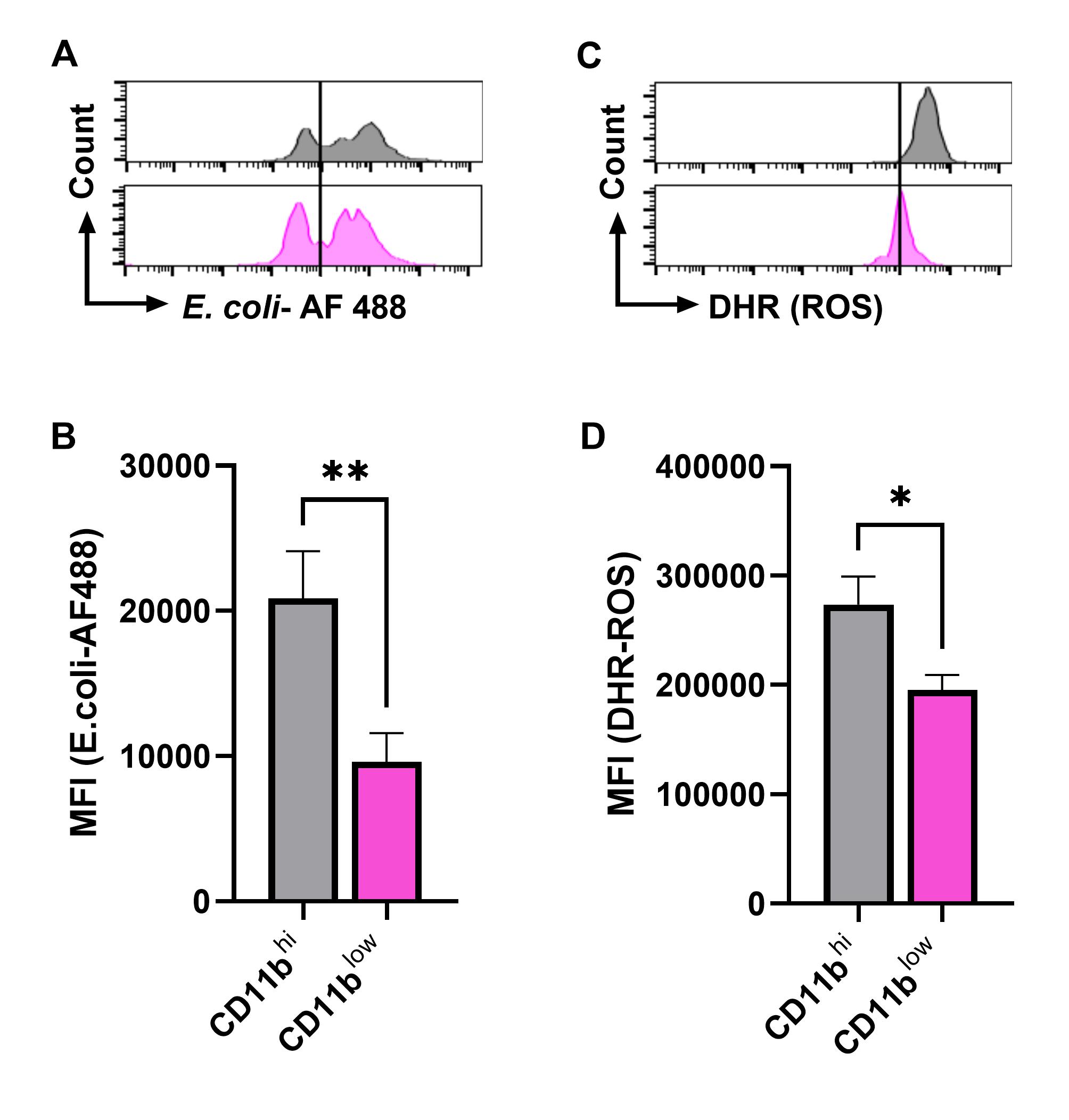

Supplement: Supplementary Figure 1 — Flow cytometric analysis of (A, B) phagocytosis (E. coli AF-488) and (C, D) oxidative burst; DHR-123 in tissue neutrophils after ex vivo fMLP stimulation. n=4; representative of 2 independent experiments, data shown as mean ± s.e.m; *p<0.05, Student’s t-test. [file Image_1.jpeg]

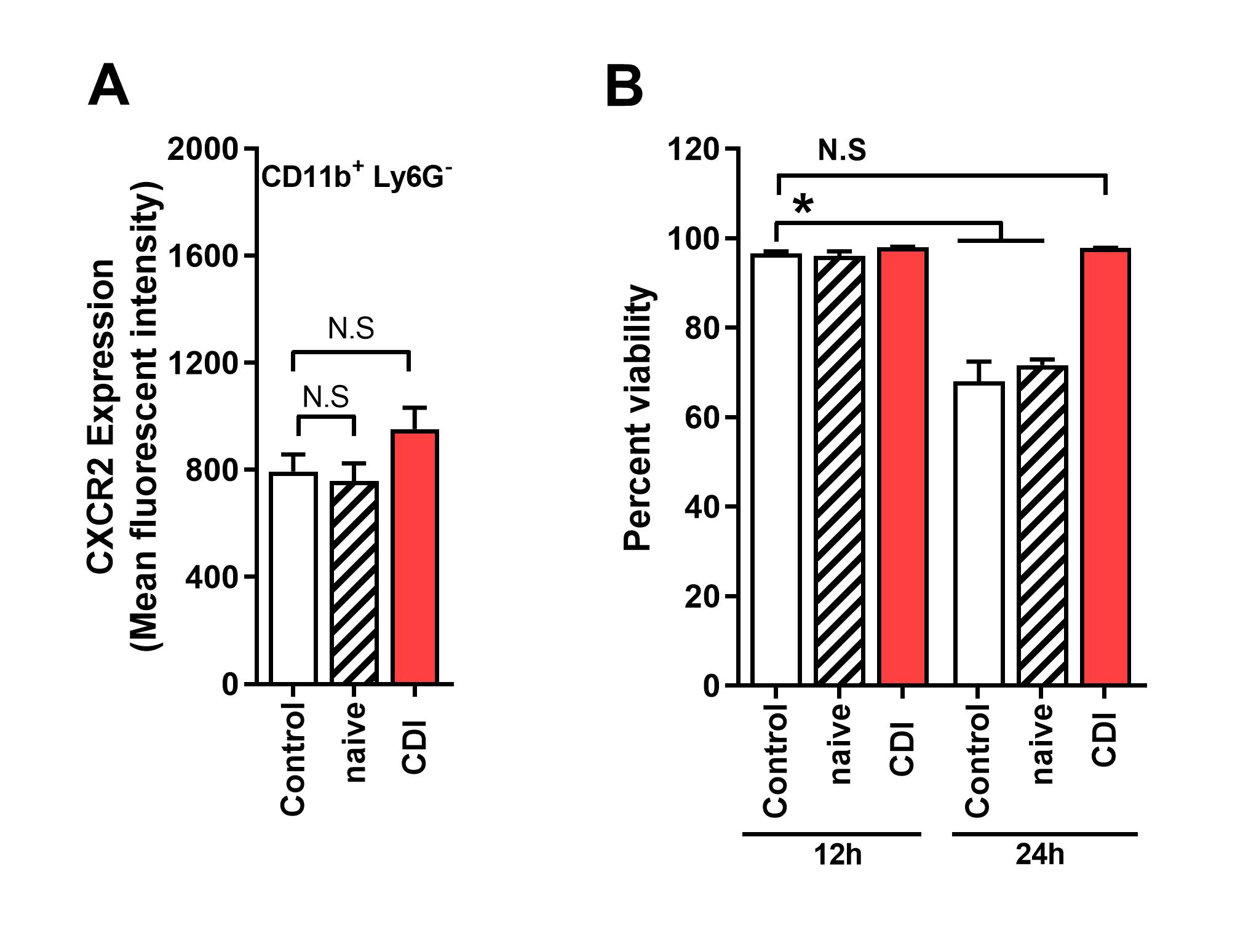

Supplement: Supplementary Figure 2 — (A) CXCR2 expression on CD11b+Ly6G- cells of bone marrow after 24h of in vitro culture in presence of plasma from C. difficile-infected RR mice. (B) Percent live neutrophils at 12h and 24h of in vitro culture. n=4-5; representative of 2 independent experiments, data shown as mean ± s.e.m; *p<0.05, Student’s t-test. [file Image_2.jpeg]

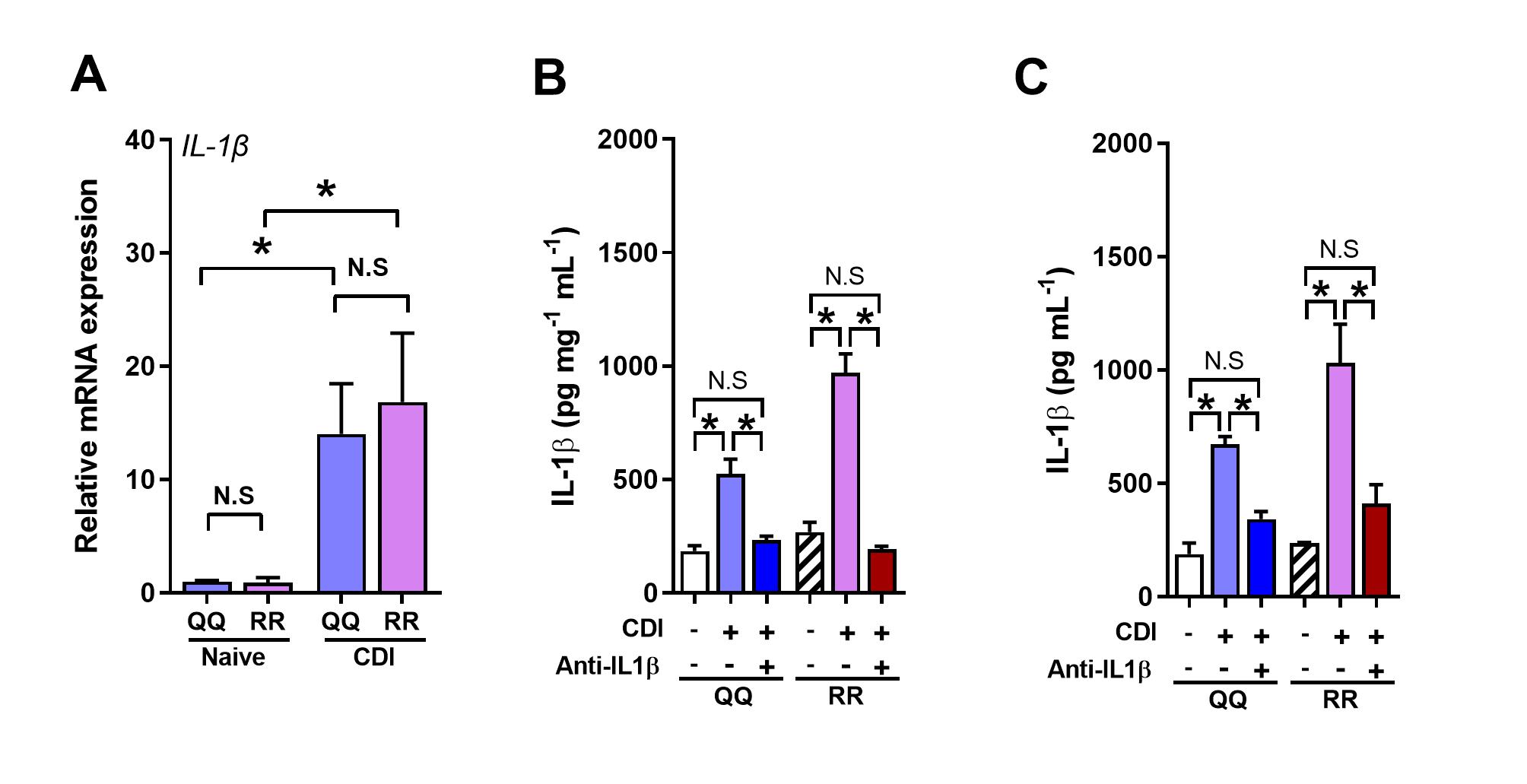

Supplement: Supplementary Figure 3 — (A) mRNA expression IL-1β in cecal tissue of naïve and C. difficile-infected (day 1) 129J QQ and RR mice. IL-1β protein concentration in (B) colonic tissue and (C) blood in QQ and RR mice after CDI and with and without anti-IL-1β antibody treatment. n=6-10 mice/group; data shown as mean ± s.e.m; *p<0.05, Student’s t-test. [file Image_3.jpeg]

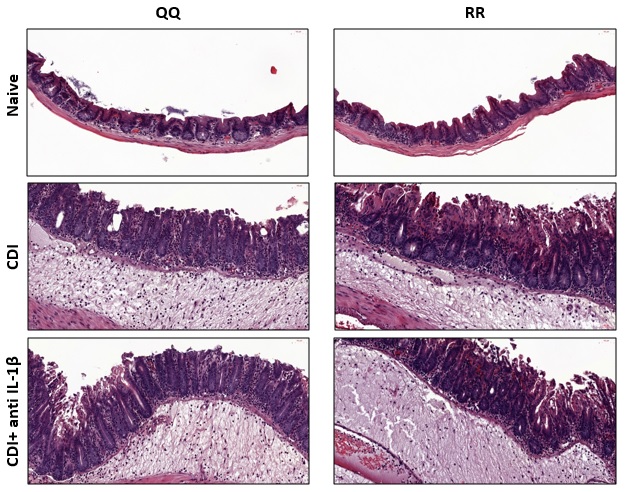

Supplement: Supplementary Figure 4 — Representative histology images of hematoxylin and eosin–stained cecal tissue sections of QQ and RR mice at day 1 of C. difficile infection. [file Image_4.jpeg]

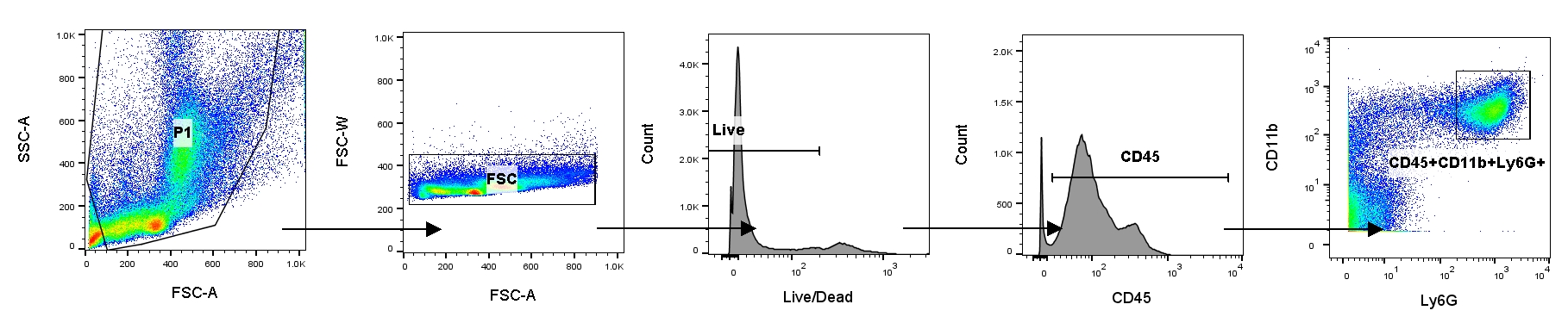

Supplement: Supplementary Figure 5 — Gating strategy for flow cytometry analysis of neutrophils. [file Image_5.jpeg]
