# Supplementary material for: Leptin Receptor q223r Polymorphism Influences Clostridioides difficile Infection-Induced Neutrophil CXCR2 Expression in an Interleukin-1β Dependent Manner
Source: Front Cell Infect Microbiol. 2021 Feb 25;11:619192. doi: 10.3389/fcimb.2021.619192 (PMC7946998; doi:10.3389/fcimb.2021.619192)
Supplement: Supplementary file 6 [file Table_1.docx]

**Supplementary Table 1**: List of antibodies used in flow cytometry

| **Antibody** | **Manufacturer** | **Clone** | **Catalogue #** |
| --- | --- | --- | --- |
| Live/Dead *Fix Far Red* | ThermoFisher Scientific | N/A | L10120 |
| CD11b - *BV650* | BD Biosciences | M1/70 | 563402 |
| Ly6G - *BV421* | BioLegend | 1A8 | 122628 |
| CXCR2 (CD182) - *FITC* | BioLegend | SA045E1 | 149608 |
| CD16/CD32 | eBioscience | 93 | 14-0161-85 |
